# Management of diabetes mellitus using medicinal plants: A review

**DOI:** 10.6026/973206300200705

**Published:** 2024-07-31

**Authors:** Anupa Sivakumar, Amardev Singh Thanu, Thirupathirao Vishnumukkala, Angu Bala Ganesh KSV, Jeevan K Shetty, Saravanan Jagadeesan, Prarthana Kalerammana Gopalakrishna

**Affiliations:** 1 Human Biology Division, School of Medicine, IMU University, Kuala Lumpur, Malaysia; 2 Department of Anatomy, Gujarat Adani Institute of Medical Sciences, Bhuj, Gujarat, India; 3 Department of Biochemistry, School of Medicine, Royal College of Surgeons in Ireland (RCSI) Bahrain, Muharraq, Bahrain; 4 Department of Anatomy, School of Medicine, Lakeside Campus, Taylor's University, Selangor, Malaysia; 5 Physiology discipline, Human Biology division, School of Medicine, IMU University, Kuala Lumpur, Malaysia

**Keywords:** Diabetes mellitus, medicinal plants, alternative therapy, *Withania somnifera*, *Trigonella foenum-graecum*, *Moringa oliefera*, *Memmordica charantia* and *Allium sativa*

## Abstract

Diabetes mellitus has a global impact affecting 422 million individuals and leading to significant health complications. This makes it a pressing global health concern. Present treatments prioritize alleviating symptoms; however, it is imperative to adopt a multitarget strategy. Herbal medicines, which have been historically employed in traditional medicine, have undergone animal experiments to assess their efficacy in reducing or preventing the disease. Known data shows that the phytochemicals found in medicinal plants have anti-hypoglycemic properties. Hence, we review the therapeutic properties of *Withania somnifera*, *Trigonella foenum-graecum*, *Moringa oliefera*, *Memmordica charantia* and *Allium sativa*.

## Background:

Diabetes mellitus (DM) is a chronic metabolic disorder of the endocrine pancreas that includes a wide range of illnesses. [1] The elevated blood glucose levels in DM result from insufficient insulin production or a diminished sensitivity to insulin. [2] As per the report by the International Diabetes Federation (IDF) 2019, 9.3 percent of adults are affected by DM, translating to approximately 463 million individuals globally. [3] If adequate preventive measures are not adequately implemented, the affected population is estimated to rise to 578 million by 2030 and 700 million by 2045. [3] Unmanaged DM can lead to the development of diabetic retinopathy, nephropathy, and neuropathy, ultimately causing dysfunction in the organ systems. [4] Insufficient insulin release from dysfunctional β-cells in the pancreas hinders the body's capacity to regulate blood glucose levels. [5] Insulin resistance (IR) increases liver glucose synthesis and lowers muscle, liver, and adipose tissue glucose uptake. [6] Oxidative stress, which involves reactive oxygen species (ROS) including superoxide, hydroxyl, peroxyl, and hydroperoxyl, and non-radical species like hydrogen peroxide, leads to DM development. [7] ROS can activate harmful pathways that accelerate DM. [6] Pharmaceutical and non-pharmacological methods treat DM. [8] Alpha-glucosidase inhibitors, sulfonylureas, biguanides, Glucagon-like peptide-1 (GLP-1) agonists, and thiazolidinediones lower glucose levels in type 2 diabetes mellitus [T2DM]. [9] Insulin is needed to treat type 1 diabetes mellitus (T1DM) and later-stage T2DM. [8] Nutrition and lifestyle changes like exercise are used under non-pharmacological approaches. [9]

## The potential of medicinal plants in diabetes mellitus management:

Since prehistoric times, traditional medicine has relied on biological treatments to treat various illnesses. [10] Modern research and evidence-based analysis have focused on evaluating herbal items or finding their active ingredients. [11] Herbal formulations contain extracts from natural herbs, fruits, and vegetables that cure various illnesses safely. [12] Several phyto-constituents and compounds from medicinal plants interact with insulin sensitivity. [10] These measures may affect pancreatic beta cell activity and treat DM [13]. This review discusses the medicinal characteristics of *Withania somnifera*, *Trigonella foenum-graecum*, *Moringa oliefera*, *Memmordica charantia*, and *Allium sativa*. There is a lot of scientific data supporting using plant extracts to treat many human and animal disorders, but their mechanisms and effects on diabetes have not been adequately studied. The species in this review were selected based on the abundant research information available and their potential to have a beneficial impact in preventing the development and control of the progression of DM and its associated problems.

## *Withania somnifera*:

*Withania somnifera* (*W. somnifera*) belongs to the Solanaceae family and is highly regarded for its significant medicinal and nutraceutical properties, making it essential in medicine. [14] *W. somnifera* has various secondary metabolites derived from its leaves, roots, and fruits, such as steroids, alkaloids, flavonoids, phenolics, saponins, and glycosides. Various components of the plant have been associated with a diverse array of preclinical experiments, encompassing cardioprotective, anticancer, antioxidant, antibacterial, antifungal, anti-inflammatory, hepato protective, anti-depressant, and hypoglycemic effects. [15] The administration of ashwagandha root extract to DM rats resulted in a reduction in the duration of immobility during the forced swim test, along with a significant increase in serotonin levels compared to those of the DM group. Compared to rats in the DM group, there was a notable rise in the overall antioxidant capacity of the brain, as well as an increase in the levels of glutathione (reduced form), superoxide dismutase, and catalase activity. Additionally, there was a significant drop in the brain's overall oxidative capacity, oxidative stress index, and malondialdehyde levels. Ultimately, it has been found that when administered at 100 and 200 mg/kg body weight, ashwagandha root extract can decrease oxidative stress and alleviate depression in the brain induced by DM. [16] Diabetic wound healing delays often occur due to increased blood vessel formation and reduced ability to respond to inflammation. Treating diabetes wounds can be effectively treated using a combination of nanoparticles (NPs) and nano-fibers. Tadalafil (TDF)-loaded nanoparticles (NPs) were incorporated into nanofibers made of polyvinyl alcohol (PVA) and *W. somnifera* extract. This combination formed the basis for creating a versatile scaffold. In a rat model with diabetes, the complete composite exhibited enhanced wound healing, reduced inflammation, and increased formation of new blood vessels. [17] Given the promising prospects of selenium nanoparticles in medicine, they play a vital role in preventing the decline and debilitation of sperm caused by chronic diseases. A study conducted by Mohammed Ali *et al.* utilized the aqueous extract of *W. somnifera* roots to prepare selenium nanoparticles (Se NPs) using a safe and non-toxic medicinal approach. The study evaluated the potential increase in antioxidant enzyme activities and Deoxyribonucleic acid (DNA) damage in sperm caused by sreptozotocin (STZ) induced diabetes in mice. The levels of superoxide dismutase (SOD) were significantly higher in the treatment groups compared to the diabetic rats. Administration of Se NP increased antioxidant enzyme activities and enhanced sperm quality in STZ-induced diabetic mice by regulating the level of reactive oxygen species. [18] *W. somnifera* studies have revealed that it is a promising plant for use as an alternative therapy for diabetes; nevertheless, more clinical trials on multitarget molecular mechanisms, safety, and dose are needed.

## *Trigonella foenum-graecum*:

*Trigonella foenum-graecum* (*Tf. graceum*), commonly known as fenugreek, belongs to the family Fabaceae. [19] The initial analysis and measurement of phytochemicals were conducted on various parts of fenugreek, including the leaves, stem, and seeds, which have shown to have a high level of active compounds such as alkaloids, terpenoids, phenols, sterols, saponins, quinones, and tannins. [20] The results of a study by Pradeep *et al.* to investigate the mechanical characteristics of the nephroprotective effect of dietary *Tf. graceum* seeds and onion on renal lesions in streptozotocin-induced diabetic rats. These nutritional modifications were observed to have normalized the balance of renal angiotensin-converting enzyme and its receptor and the effect of *Tf. graceum* and onion in the diet significantly reduced nitric oxide levels, N-acetyl-β-d-glucosaminidase activity, and the polyol pathway metabolites-the *Tf. graceum* and onion combination in diet also decreased the expression of kidney injury molecule-1. They improved the excretion of urine indicators such as nephrin, podocin, and podocalyxin, which are associated with damage to podocytes and diabetes-induced structural damage in the kidney. [21] Hydroalcoholic extract from leaves of *Tf. graceum*, when administered at a dosage of 500 mg/kg, exhibited enhanced neovascularisation, accelerated re-epithelialization, improved control of fibroblast and macrophage presence in the wound bed, and moderate collagen synthesis observed on the wound healing capabilities of diabetic rats. [22] Combined administration of 200 mg/kg of the extract and metformin to diabetic mice showed reduced levels of malondialdehyde (MDA) and nitric oxide (NO) metabolites with increased levels of thiols, superoxide dismutase (SOD), and catalase (CAT). The hydroalcoholic extract from the *Tf. graceum* seed, when administered along with metformin, effectively alleviated cognitive impairments associated with diabetes by partially reducing oxidative damage in the brain, exhibiting its therapeutic benefits. [23] The beneficial effects of *Tf. graecum* seed extract on diabetes-induced rats also improved performance in behavioral tasks like Morris water maze and T-maze, which was associated with lower blood glucose levels, reduced oxidative stress in the hippocampus, and lesser neuronal loss observed in the CA1 and CA3 regions of the hippocampus. [24] In a study by Luo *et al.* on the integrated network pharmacology and molecular docking show the active components of *Tf. graceum* and their potential mechanisms in combating diabetes. The results demonstrated that *Tf. graceum* is biologically benign and effectively enhances glucose uptake by IR-HepG2 cells, while the pathway enrichment study revealed antidiabetic benefits of *Tf. graceum* were regulated by the AGE-RAGE and Nuclear factor kappa-light-chain-enhancer of activated B cells (NF-κB) signaling pathways, which primarily relate to the protection of β-cells, the suppression of inflammation, and the reduction of oxidative stress activity of the extract. [25] Various preclinical studies have shown that *Tf. graceum* has the potential to be an alternative treatment for DM. However, more thorough research is required to evaluate the long-term effects and their outcome in multiple organ involvement in diabetes. Additional clinical trials are also required to design the dosage for future use.

## *Moringa oliefera*:

Moringa oleifera (*M. oleifera*) belongs to the Moringaceae family, order Capparidales, class Magnoleopsida. [26] *M. oleifera*, is used in India since the 18th century BC, has numerous medicinal uses in the Ayurvedic and Unani systems. Its parts, including root, bark, gum, leaf, fruit, flowers, seeds, and seed oil, treat skin infections, anemia, asthma, bronchitis, diarrhea, joint pain, rheumatism, gout, diabetes, conjunctivitis, and hemorrhoids. [27] *M. oleifera* leaves have therapeutic effects due to their bioactive components, including trace metal ions, vitamins, alkaloids, carotenoids, and essential amino acids. [26] Due to their antioxidant properties, *M. oleifera* plants garner increasing attention for treating diabetes mellitus. Tumour Necrosis Factor alpha (TNF alpha) and Interferon gamma (IFN-γ) produced by natural killer (NK) cells were recently efficiently inhibited in experimental mice that had been given streptozotocin-induced Type 1 diabetes (T1DM)by 800 mg/kg body weight of MO plus 615 mg/kg BW albumin. In people with type 1 diabetes, these synergies may reduce or suppress TNF-α and IFN-γ levels. This may be because flavonoids block the tumor necrosis factor-alpha-converting enzyme (TACE), which lowers the amount of TNF-α produced by NK cells. [28] With a focus on its antidiabetic and antioxidant qualities, an aqueous extract of *M. oleifera* leaves was included in the study to treat diabetic albino rats induced by STZ. Reduced levels of glutathione, malondialdehyde, and fasting plasma glucose were also observed, along with significant improvements in the extract's capacity for reversing islet cell histopathological damage. [29] There was no significant difference in HbA1C or fasting plasma glucose (FPG) levels in a study by R Taweerutchana that looked at the effects of *M. oleifera* leaf capsules on glucose control in type 2 diabetes patient's naïve to therapy. Still, Moringa oleifera leaf showed a tendency towards lowering blood pressure. The study's short duration necessitates a more extensive and extended study to understand the full impact of *M. oleifera* leaf on FPG. [30] A study involving subjects with prediabetes found that *M. oleifera* leaves as a food supplement led to favorable changes in glycemia markers compared to a placebo. The study revealed significant differences between groups in fasting blood glucose and glycated hemoglobin rate, with opposite directions during the intervention. However, no significant changes in microbiota, hepatic, renal function markers, or appetite-controlling hormones suggested its function as a natural antihyperglycemic agent. The study had its limitations, such as moderately raised mean glycemia prior to the intervention and the exploratory nature of potential factors influencing response to the intervention. Hence, future trials should investigate the parts of *M. olifera* other than the leaves for activity. [31] A study by AL Al-Malki investigated the antidiabetic effect of Moringa seed powder on streptozotocin-induced diabetes in male rats. The rats were divided into four groups, and the diabetic group showed increased lipid peroxide, interleukin-6[IL-6], and antioxidant enzyme levels. However, treatment with Moringa seeds powder improved these parameters. It restored normal histology in kidney and pancreas tissues compared to the diabetic positive control group due to its content of antioxidant compounds such as glucomoringin, phenols, and flavonoids. [32] Studies on Moringa oleifera have shown that it may impact diabetes; however, human clinical trials are required to confirm results and evaluate long-term effects, safety, efficacy, and appropriate dosages.

## *Momordica charantia*:

*Momordica charantia* (*M. charantia*) is a tropical and subtropical vine belonging to the Cucurbitaceae family. It is widely cultivated throughout Asia, Africa, and the Caribbean for its therapeutic benefits. [33] *M. charantia* has been used for millennia in traditional medicine to treat a variety of conditions, including diabetes mellitus, hypertension, obesity, cancer, bacterial and viral infections. [34] It also includes other substances that may benefit health, including alkaloids, triterpenoids, polypeptides, and lectins. Owing to its intriguing bioactive components and lengthy history of traditional use, *M. charantia* has drawn interest as a possible substitute treatment for diabetes mellitus. [35] It is rich in substances, including charantin and polypeptide-p. It has been shown to mitigate insulin dependency in individuals with type 2 diabetes by promoting glucose transfer into cells and triggering Adenosine monophosphate-activated protein kinase (AMPK). This enzyme controls cellular energy balance. Bitter gourd may improve insulin secretion in T2DM patients by stimulating pancreatic β-cells, reducing oxidative stress, and inhibiting glucose absorption. Momordicosides in bitter gourd stimulate insulin release, while antioxidants protect β-cells from oxidative damage. Additionally, bitter gourd lectins inhibit α-glucosidase enzymes, slowing down carbohydrate digestion and gradually increasing blood glucose levels after meals. [36] Oxidative stress plays a role in the onset and progression of diabetes. The antioxidants found in bitter gourd, including vitamin C, vitamin E, and flavonoids, assist in lowering oxidative stress and inflammation in the body [35]. Jiang *et al.* found that MC saponins (MCS) increased SOD and CAT enzyme activity and decreased MDA levels in rats' pancreatic and liver tissue, preventing oxidative stress damage after four weeks of administration of MCS. [37] A study by Chokki *et al.* evaluated the antioxidant and enzyme inhibitory effects of *M. charantia* and *M. lucida* leaves, focusing on their antidiabetic activity, using micro-dilution techniques and DPPH free radical scavenging activity. The α-amylase inhibition assay and β-glucosidase inhibition assay were conducted using the 3,5-dinitro salicylic acid procedure and p-nitrophenyl-β-D-glucopyranoside substrate, respectively, using HPLC. Results showed that both plants contain polyphenol compounds, with dichloromethane and ethyl acetate extracts showing good α-amylase inhibitory activity. *M. lucida* dichloromethane extract showed the highest inhibitory capacity of β-glucosidase activity. These results imply possible application in traditional medical applications. [38] Animal experiments show that *M.charantia* can treat Type 2 Diabetes by improving insulin signal transduction disorder. Ethanol extract in diabetic rats reduces SOCS-3 expression, improves IRS-1/PI3K signal transduction, and increases liver glycogen content. [39] The study by Yang *et al.* aimed to explore the potential of BM in preventing insulin resistance and diabetes in obese and diabetic rats and to understand the mechanism behind its amelioration. The study compared rats on a high-fat diet with 1% BM and 3% BM. Results showed no changes in body weight or food intake, improved glucose tolerance and insulin sensitivity, down-regulated proinflammatory cytokines, and decreased NF-κB activation in the liver and muscle. The study found that BM supplementation increased phospho-insulin receptor substrate-1 and phospho-Akt levels and decreased phospho-NF-κB and JNK levels in liver, muscle, and epididymal fats. [40] Furthermore, it has been discovered that MC juice can boost skeletal muscle's absorption of glucose via the PI3K pathway. [41] Research has shown that *M. charantia* has potential antidiabetic effects, including the capacity to reduce blood glucose levels and enhance insulin sensitivity. However, it is essential to highlight that the bulk of this research has been conducted on animals or in vitro, and there is a scarcity of well-designed clinical trials in humans to back up these findings. As a result, while preliminary research on *M. charantia* is encouraging, additional studies are needed to determine its efficacy and safety in humans.

## *Allium sativum*:

*Allium sativum* (*A. sativum*), commonly known as garlic, is a member of the Alliaceae family. It is widely produced and used globally as a spice, additive, and medicinal herb. Due to multiple biologically active components, garlic is well-suited for therapeutic purposes. [42] In rats with diabetes induced by STZ, administering Vernonia amygdalina combined extract (VAAS) significantly reduced fasting blood glucose levels and increased body weight. After DM induction, there was a significant reduction in the levels of superoxide dismutase (SOD), catalase (CAT) activity, and reduced glutathione (GSH) concentration. However, there was a significant increase in these levels following VAAS therapy. However, after DM induction, there was a notable rise in levels of malondialdehyde (MDA), urea, alanine transaminase (ALT), aspartate transaminase (AST), and alkaline phosphatase (ALP). These levels were then reduced after VAAS treatment in these male Wistar rats, who also exhibited signs of enhanced tissue architecture in their liver and kidney following injury caused by STZ. [43] The Allium sativum Essential Oil (ASEO)'s antidiabetic capacity was assessed by investigating yeast cells' glucose absorption at various glucose concentrations (5mM, 10mM, 25mM). The findings indicated that the ASEO enhanced glucose uptake across the yeast cell membrane. However, future studies may look into separating and identifying significant functional aspects to determine the observed phenomenon. [44] Xie *et al.* studied using an animal model to investigate the antihyperglycemic effect and potential hypoglycemic mechanism of garlic (*Allium sativum L.*) polysaccharide (GP). When intragastric administration was done with GP, it effectively reduced the symptoms of excessive hunger and thirst in mice with diabetes. The fasting blood glucose (FBG) of the high-dose GP (DGH) group was considerably 42% lower than that of the diabetic model (DC) group, suggesting a pronounced hypoglycemic effect. GP can regulate hepatic glycogen metabolism by modulating the levels of phosphoenolpyruvate carboxykinase (PEPCK), glycogen synthase (GS), and glucokinase (GK). Due to their structure, 20.7% of the side chains in GP, which had a low relative molecular weight (MW) of 2.0 kDa, were responsible for the hypoglycemic effect. This implies that GP could enhance the effectiveness of the intervention. [45] Subsequent research examined the influence of non-fermented (NFG) and fermented (FG) garlic on insulin levels. These drugs function by delaying the assimilation of carbohydrates, hence reducing blood glucose levels. The study's findings indicate that FG and NFG extract effectively lower blood sugar levels in Wistar rats when administered at 75 mg/kg BW, showcasing its anti-inflammatory and antioxidant properties. [46] An RCT by Memon AR *et al.* investigated the impact of a blend of Allium sativum and Olea europaea oil on abnormal lipid profiles in individuals with type 2 diabetes mellitus (T2DM). This study employed a sample size of 160 individuals, comprising both males and females aged 40 to 60, who were diagnosed with both type 2 diabetes mellitus (T2DM) and dyslipidemia. The participants were evenly distributed into two groups. Throughout the experiment, blood samples were obtained at three distinct stages to simplify the analysis of lipid profiles. Analysis indicated that the mean concentrations of blood cholesterol, triglycerides (TGs), and low-density lipoprotein (LDL) decreased in both groups following 3 and 6 months of treatment. Antioxidants in the investigated substances may explain the observed antihyperlipidemic action. [47]

## Conclusion:

Diabetes is a metabolic condition that is currently managed with drugs. Traditional treatments have produced beneficial outcomes when extracts from medicinal plants are used. These plants have the potential to be used in the development of alternative therapies that enhance blood glucose levels and prevent complications in diabetes mellitus. However, further research is needed on the clinical trials in humans on dosage and efficacy to use as an alternative for diabetes mellitus.

## References

[1] American Diabetes Association (2010). Diabetes Care..

[2] Silva JAD (2018). BMC Public Health..

[3] Saeedi P (2019). Diabetes Res Clin Pract..

[4] Forbes JM, Cooper ME. (2013). Physiol Rev..

[5] Banday MZ (2020). Avicenna J Med..

[6] Samuel VT, Shulman GI. (2016). J Clin Invest..

[7] Caturano A (2023). Curr Issues Mol Biol..

[8] Weinberg Sibony (2023). Int J Mol Sci..

[9] Garedow AW (2023). Sci Rep..

[10] Hardy K (2021). Rev Bras Farmacogn..

[11] Yuan H (2016). Molecules..

[12] Thirupathirao Vishnumukkala (2024). International Journal of Ayurveda and Pharma Research..

[13] Ali Hussain HE. (2002). Indian J Clin Biochem..

[14] Mikulska P (2023). Pharmaceutics..

[15] Saleem S (2020). Iran J Basic Med Sci..

[16] Hashem H.A (2023). Journal of Advanced Veterinary Research,.

[17] Elsherbini AM (2023). Nanomedicine (Lond)..

[18] Mohammed Ali IA (2023). Appl Sci..

[19] Albaker WI (2023). J Pers Med..

[20] Salam SGA (2023). Sci Rep..

[21] Pradeep SR (2019). Nutrition..

[22] Salimabad F (2023). J Wound Care..

[23] Bafadam S (2019). Horm Mol Biol Clin Investig..

[24] Kodumuri PK (2019). J Basic Clin Physiol Pharmacol..

[25] Luo W (2023). J Cell Mol Med..

[26] Mbikay M (2012). Front Pharmacol..

[27] Vergara-Jimenez M (2017). Antioxidants (Basel)..

[28] Vargas-Sánchez K (2019). Nutrients..

[29] Yassa HD, Tohamy AF. (2014). Acta Histochem..

[30] Taweerutchana R (2017). Evid Based Complement Alternat Med..

[31] Gómez-Martínez S (2021). Nutrients..

[32] Al-Malki AL, El Rabey HA. (2015). Biomed Res Int..

[33] Grover JK, Yadav SP. (2004). J Ethnopharmacol..

[34] Prasad V (2006). J Herb Pharmacother..

[35] Çiçek SS. (2022). Front Pharmacol..

[36] Sun K (2023). J Ethnopharmacol..

[37] Xu B (2022). Molecules..

[38] Chokki M (2020). Foods..

[39] Ma C (2017). Pharm Biol..

[40] Yang SJ (2015). J Nutr Biochem..

[41] Ahmed I (2004). Mol Cell Biochem..

[42] Baltzi E (2024). Biomed Rep..

[43] Asante DB, Wiafe GA. (2023). J Diabetes Res..

[44] Eidi A (2006). Phytomedicine..

[45] Shang A (2019). Foods..

[46] Sivamaruthi BS (2018). Nutrients..

[47] Memon AR (2022). J Taibah Univ Med Sci..

